# No-Cook Process for Ethanol Production Using Indian Broken Rice and Pearl Millet

**DOI:** 10.1155/2012/680232

**Published:** 2012-01-31

**Authors:** Vipul Gohel, Gang Duan

**Affiliations:** ^1^Grain Processing, Genencor, a Danisco Division, Danisco (India) Pvt. Ltd., Plot no. 46, Roz-Ka-Meo Industrial Area, Sohna, Tehsil NUH, District-Gurgaon 122 103 Sohna, India; ^2^Grain Processing, Genencor (China) Bio-Products Co. Ltd., 102, Mei Li Road, Wuxi New District 214028 Wuxi, China

## Abstract

No-cook process using granular starch hydrolyzing enzyme (GSHE) was evaluated for Indian broken rice and pearl millet. One-factor-at-a-time optimization method was used in ethanol production to identify optimum concentration of GSHE, under yeast fermentation conditions using broken rice and pearl millet as fermentation feedstocks. An acid fungal protease at a concentration of 0.2 kg per metric ton of grain was used along with various dosages of GSHE under yeast fermentation conditions to degrade the grain proteins into free amino nitrogen for yeast growth. To measure the efficacy of GSHE to hydrolyze no-cook broken rice and pearl millet, the chemical composition, fermentation efficiency, and ethanol recovery were determined. In both feedstocks, fermentation efficiency and ethanol recovery obtained through single-step no-cook process were higher than conventional multistep high-temperature process, currently considered the ideal industrial process. Furthermore, the no-cook process can directly impact energy consumption through steam saving and reducing the water cooling capacity needs, compared to conventional high-temperature process.

## 1. Introduction

Food and energy security has always been key priorities due to various reasons. This is due to their limited availability and increasing demand with ever increasing population [1–3]. At the same time, the demand for ethanol has been increasing since it is considered to be an alternative transportation energy source in addition to its use for recreational consumption [4, 5]. Considerable attention has been given to first ethanol production from various available sugar substrates such as molasses, sugar cane juice [6]; starchy materials like rice, millet, corn, sorghum, wheat, potato, cassava [7–10]; cellulosic materials as second-generation ethanol [11]. Pearl millet, broken rice, and sorghum are the major starchy materials used by Indian ethanol producers not only for the production of potable alcohol [12] but also for fuel purposes (http://www.icrisat.org/text/research/grep/homepage/sgmm/chapter12.pdf). Moreover, Indian ethanol producers use these raw materials based on their availability and cost since these are seasonal grains [12, 13].

The increasing price of crude oil and other fossil fuels has increased the interest in alternative fuel sources around the world [14, 15]. Fuel alcohol production from starch materials needs constant process improvement for meeting the economic payback by lowering expensive energy consumption and improvement in fermentation efficiency in order to be considered as a viable alternative to fossil fuel. At present, the production cost for ethanol is INR 20 to 23 per liter from molasses-based ethanol plants (1.0 INR = 0.0225683 USD), which is slightly higher than the cost in Brazil using molasses (INR 14 to 16 per liter) [16]. Indian ethanol producers are seeking technological alternatives that would lower the cost and provide higher margin in order to compete with gasoline and other fossil fuels. Utility consumption involves energy, electricity, water cooling, and heating. Water and energy (steam and cooling is generated with water) are the most extensively used commodities in process industries. Water scarcity and environmental regulations on water effluents are a major concern nowadays. In particular, grain-based bioethanol plants are water and energy intensive [17, 18]. A molasses-based plant with 100 kL per day capacity will require 450 kWH power, 1620 to 1800 kL water per day for molasses dilution; and cooling water requirement will be 1080 kL per day. For a plant of such capacity, 2.0 to 2.3 MT of steam for 1.0 kL of ethanol production is required. In India, due to limited availability of molasses, molasses alone is not sufficient to meet the growing ethanol needs of the country, especially for use as a biofuel. Furthermore, the government of India is aggressively promoting the concept of blending petrol (gasoline) with ethanol to reduce dependence on petrol, and about 500 million liters of ethanol would be required every year, even if 10% ethanol is blended with gasoline (http://www.gujagro.org/agro-food-processing/molasses-base-alcohol-34.pdf). Thus, a number of distilleries have started converting their molasses-based plants into cereal-grain based ethanol production [5]. However, ethanol production cost is INR 23 to 28 per liter with grain-based technology compared to molasses-based technology. The major factors for such higher production cost are considered to be raw materials, steam, electric power, and cooling water required for enzymatic liquefaction; saccharification; fermentation; distillation process. Moreover, depending on the technology, and raw material selection by industries, utility consumption will vary (http://ejournal.icrisat.org/mpii/v3i1/impi1.pdf) [16].

Most biological processes are based on the conversion of starchy materials of grain or cereals into glucose and, in turn, its subsequent conversion into ethanol; which consists of three steps, starch liquefaction (80 to 125°C), saccharification (55 to 65°C), and fermentation (32 to 35°C) of sugar to ethanol [7]. Advanced developments have further reduced one enzymatic process step of separate saccharification (55 to 65°C) since the availability of energy or resource or utility is a major concern to the industry as these factors directly impact production costs [19]. The improved biological process of starch materials conversion is liquefaction and simultaneous saccharification and fermentation (SSF) a process in which the saccharifying enzyme further hydrolyzes the liquefied starch into fermentable sugars at yeast fermentation conditions and simultaneously enables the fermentation of sugars to ethanol [19]. However, SSF has not significantly impacted energy consumption because liquefaction of starch takes place at high temperatures ranging from 80 to 125°C [3, 20, 21] requiring enormous amounts of steam and an efficient water-based cooling system to bring down the temperature from 80–125°C to 32–35°C for SSF process [19, 22].

The granular starch hydrolyzing enzyme (GSHE) developed by GENENCOR, a Danisco Division, was used to hydrolyze no-cook starch directly to fermentable sugars under yeast fermentation conditions without using steam. This process has the additional advantages of improving the efficiency of starch conversion into ethanol due to reduced sugar loss that is inevitable with a high-temperature cooking process and producing less biomass due to reduced stress of yeast. The no-cook process enables all these biological processes in a single step without requiring any steam to cook the starchy materials [23, 24]. It is also known that ethanol fermentation based on “granular starch hydrolysis” is associated with better recovery of value-added products compared to the traditional jet-cooking fermentation or conventional process [2, 3, 25].

Moreover, the chemical and nutritional quality of fermentation feedstocks of starchy substrates like broken rice and pearl millet varies considerably from one geographic region to another, and this may be attributed to genetic factors; environmental influences; fertilizer treatments; degree of milling; storage conditions. It has been reported that these factors also impact the ethanol yield [3, 26].

Thus, the objective of the present study was necessary to evaluate the substrate composition prior to the ethanol production through a no-cook process and determine the efficiency of GSHE under yeast fermentation conditions using Indian broken rice and pearl millet as fermentation feedstocks.

## 2. Materials and Methods

### 2.1. Enzymes, Reagent and Chemicals

Granular starch hydrolyzing enzyme (GSHE) is an enzyme cocktail containing fungal alpha amylase and a glucoamylase that work synergistically to hydrolyze granular starch to glucose (STARGEN 002, activity minimum 570 GAU/gm, one glucoamylase unit [GAU] is the amount of enzyme that will liberate one gram of reducing sugars calculated as glucose per hour from soluble starch substrate under the assay conditions, http://www.genencor.com/); FERMGEN (acid fungal protease, activity minimum 1000 SAPU/gm, the activity of FERMGEN protease is expressed in spectrophotometric acid protease units [SAPU], one SAPU is the amount of enzyme activity that liberates one micromole of tyrosine per minute from a casein substrate under conditions of the assay, http://www.genencor.com/); SPEZYME FRED (alpha-amylase, activity minimum 17,400 LU/gm, one liquefon unit [LU] is the measure of the digestion time required to produce a color change with iodine solution, indicating a definite stage of dextrinization of starch substrate under specified conditions, http://www.genencor.com/); OPTIDEX L-400 (glucoamylase, activity minimum 350 GAU/gm, one glucoamylase unit [GAU] is the amount of enzyme that will liberate one gram of reducing sugars calculated as glucose from a soluble starch substrate per hour under the specified conditions of the assay, http://www.genencor.com/) were obtained from GENENCOR a Danisco Division. Active dry yeast from AB Mauri India Pvt. Ltd (MIDC −415 722, India) and urea from Merck (ML7M573074; 60848605001730) were purchased. Industrial grade Indian broken rice and pearl millet grains were purchased from the local market.

### 2.2. Milling of Indian Broken Rice and Pearl Millet

Indian broken rice and pearl millet were milled using laboratory milling grinder (Milcent, Anand, Gujarat-India). A sieve analysis showed that 90% of the resulting flour had a particle size that passed through U.S. standard 40 mesh-sieves.

### 2.3. Chemical Composition of Indian Broken Rice and Pearl Millet

Oil, tannin, total free P_2_O_5_, crude fibers and fat (lipid) contents in broken rice and pearl millet were analyzed as described in AOAC 18th EDN: 2006.

### 2.4. Soluble Glucose and Fructose Content

Soluble glucose and fructose in Indian broken rice and pearl millet flour were extracted in water. For this, 1.0 gm of Indian broken rice or pearl millet flour (dry basis) was dissolved in 99 mL of water and mixed for 1 hr at ambient temperature. The sample was then analyzed by HPLC (Agilent Isocratic system 1200, USA) on an Aminex Column HPX-87H (catalogue number 1250140, Bio-Rad) at 60°C with a mobile phase of 0.01 N sulfuric acid at a flow rate of 0.7 mL/min. A standard containing glucose (0.5%) and fructose (0.5%) was used to identify and quantify the products:

(1) $$\begin{array}{cc} & \%\;\text{soluble glucose}\\ & \;=\left(\frac{\%\;\text{glucose}}{100}\right)\\ & \;\;\times \left(\frac{100}{\left(\text{grain weight, gm}\right)\times \;\left(\%\;\text{dry solids}/100\right)}\right)\times 100,\\ & \%\;\text{soluble fructose}\\ & \;=\left(\frac{\%\;\text{fructose}}{100}\right)\\ & \;\;\times \left(\frac{100}{\left(\text{grain weight, gm}\right)\times \;\left(\%\;\text{dry solids/}100\right)}\right)\times 100. \end{array}$$

### 2.5. Starch Content

For analyzing the starch content in Indian broken rice and pearl millet grain, the grains were milled so that less than 10% of particles were retained on U.S. 40 mesh-sieve. The grain flour was hydrolyzed using an enzymatic method where alpha-amylase, SPEZYME FRED and glucoamylase, OPTIDEX L-400 were used for liquefaction and saccharification process, respectively. The end product glucose was further analyzed by HPLC (Agilent Isocratic system 1200, USA) as described in Section 2.4:

(2) $$\begin{array}{cc} & \%\;\text{total}\;\text{glucose}\\ & \;=\left(\frac{\%\;\text{glucose}}{100}\right)\\ & \;\;\times \left(\frac{100}{\left(\text{grain weight, gm}\right)\times \;\left\{\%\;\text{dry solids/}100\right\}}\right)\times 100,\\ & \%\;\text{starch}\\ & \;=(\%\;\text{total}\;\text{glucose}\;\text{in}\;\text{grain}\;\text{sample}\;\text{from}\;\text{Et}\\ & \;\;-\text{\%}\;\text{soluble}\;\text{glucose}\;\text{in}\;\text{grain}\;\text{sample}\;\text{from}\;\text{We})\times 0.9, \end{array}$$

where Et is enzyme-treated sample and We is water extracted sample.

### 2.6. Protein Content

The protein content in Indian broken rice and pearl millet feedstocks was estimated by the Kjeldahl's Method (IS 7219:1973 (Reaff. 2005)).

### 2.7. Optimization of GSHE Concentration for Ethanol Production Based on CO_**2**_ Released under the Yeast Fermentation Conditions Using Indian Broken Rice and Pearl Milllet

One-factor-at-a-time optimization method was used to identify optimum concentration of GSHE, under the yeast fermentation conditions using Indian broken rice and pearl millet separately as fermentation feedstocks.

Slurry of 25% DS (dry solid) of Indian broken rice and pearl millet flour as fermentation feedstocks was prepared in a 1-liter flask separately by adding the RO water. The pH of the slurry of Indian broken rice and pearl millet flour was adjusted to 4.5 using 6 N H_2_SO_4_. A one-factor-at-a-time optimization method was used to identify the optimum concentration of Granular Starch Hydrolyzing Enzyme (GSHE), STARGEN 002, under yeast fermentation condition using Indian broken rice and pearl millet as fermentation feedstocks. The STARGEN 002 (GSHE) concentration of 1.5, 2.0, 2.5 and 3.0 kg per MT of grain was used for both the grains. Concurrently FERMGEN (proteases), 0.2 kg per MT of grain; urea, 400 ppm; and active dry yeast, 0.25% were added. The flask was covered with a sterile plug and its initial weight recorded before incubating at 32 ± 2°C in a rotary shaker at 300 rpm. The flask weight (gm) and medium pH was measured at 24 hr intervals of fermentation process to calculate the ethanol production (%, w/w) based on weight-loss or CO_2_ released by using following calculations.

(3) $$\begin{array}{cc} & 1\;\text{MT}\;\text{of}\;\text{grain}\;\text{to}\;\text{ethanol}\;\text{in}\;\text{Lit}\\ & \;\;=\left(\frac{1000}{\text{grain}\;\text{weight},\;\text{gm}\times \left\{\text{initial}\;\text{slurry}\;\text{weight},\;\text{gm}-24\;\text{hr}\;\text{intervals}\;\text{slurry}\;\text{weight},\;\text{gm}\right\}}\right)\times \left(\frac{0.789}{44}\right),\\ & \text{ethanol}\;\text{production}\;\left(\text{\%},\;\text{w}/\text{w}\right)\;\text{based}\;\mathrm{on}\;{\text{CO}}_{2}\;\text{released}=\frac{\text{total}\;\text{grain}\;\text{used},\;\text{gm}\times 1\;\text{MT}\;\text{grain}\;\text{to}\;\text{ethanol}\;\text{in}\;\text{L}}{24\;\text{hr}\;\text{intervals}\;\text{Slurry}\;\text{weight},\;\text{gm}\times 0.789}. \end{array}$$

### 2.8. Ethanol Yield, Residual Starch, and Sugar Analysis

The fermentation slurry was distilled at 80°C by using Soxhlet's apparatus (Ambassader; B.P. Industries, Delhi-India) in 72 hr of yeast fermentation process. The distilled ethanol (% v/v at 20°C) was measured by using an alcometer. At the same time, residual sugar in the fermented slurry was estimated by the Lane and Eynon's method [27] and residual starch was determined using an enzymatic method with alpha-amylase, SPEZYME FRED and glucoamylase, OPTIDEX L-400 used for the liquefaction and saccharification processes, respectively [28]. The total sugar formation by enzymatic method was also estimated by Lane and Eynon's method [27], 1% glucose was used as the standard.

### 2.9. Ethanol Recovery and Fermentation Efficiency

After laboratory distillation of the fermented slurry, ethanol recovery (liter per MT of grain) and fermentation efficiency (%) were further calculated by using the following equations, respectively:

(4) $$\;\begin{array}{cc} & \text{ethanol}\;\text{recovery}\;\left(\text{L}/\text{MT}\text{of}\;\text{grain}\right)\\ & \;=\frac{\text{total}\;\text{slurry},\text{L}\;\times \text{ethanol}\;\text{\%}\;\text{v}/\text{v}\;\text{at}\;20^{{}^{\circ}}\text{C}}{\text{total}\;\text{grain},\;\text{MT}}, \end{array}$$

(5) fermentation  efficiency  (%) =total  slurry,  gm×ethanol  %,  v/v  at  20C°×100  total  grain,  gm×%  starch×1.11×0.646.

All the experiments were done in triplicates and the values are represented statistically in analysis of variance (ANOVA) form.

## 3. Results and Discussion

### 3.1. Composition of Indian Broken Rice and Pearl Millet

The chemical and nutritional quality of fermentation feedstocks of broken rice and pearl millet was essential to evaluate the substrate composition prior to the ethanol production through a no-cook process. Composition contents (%, dry basis) of 68.45 starch; 0.34 soluble glucose; 0.08 soluble fructose; 9.38 protein; 1.76 fat (lipid); 0.72 P_2_O_5_; 2.51 crude fibers; 0.12 tannin; 3.43 oil; 3.23 others, (non-starch-polysaccharide, minerals, ash content, etc.) were found in Indian broken rice whilst 60.00 starch; 0.63 soluble glucose; 0.45 soluble fructose; 8.34 protein; 5.90 fat (lipid); 1.37 P_2_O_5_; 4.18 crude fibers; 0.28 tannin; 5.48 oil; 2.91 others were observed in Indian pearl millet. It has been reported that cooking at higher temperature in conventional processes causes the chemical components of grains to be inactivated or become toxic to the yeast, which further interferes with the ethanol yield [3, 26] (http://www.afripro.org.uk/papers/Paper08Hamaker.pdf). Moreover, it has also been reported that following a no-cook process can impact their value in distillers dried grains with solubles (DDGS) quality or alternatively, these chemical components can be further converted into monomers by using an enzymatic process to add nutrients that facilitate yeast growth [3, 29]. With this aim, acid fungal protease (FERMGEN) along with various dosages of GSHE (STARGEN 002) was used in the initial stage of the granular starch hydrolysis process under yeast fermentation conditions. This acid fungal protease hydrolyzes the proteins present in the grains into amino acids, peptides, and free amino nitrogen (FAN) essential for yeast growth. Furthermore, it has been reported that protease plays a key role not only in hydrolyzing the protein matrices in the kernel that binds the various fractions, which releases “hard-to-hydrolyze” starch, but also in accelerating ethanol production rates and higher ethanol yield for grain based substrates as compared to those without protease [30]. While using acid fungal protease (FERMGEN) along with various concentrations of GSHE (STARGEN 002) for Indian broken rice and pearl millet feedstocks separately under yeast fermentation conditions, optimum ethanol production was observed at the 60 hr fermentation cycle.

### 3.2. Optimization of GSHE Concentration for Ethanol Production Based on CO_**2**_ Released

**Figure 1:**
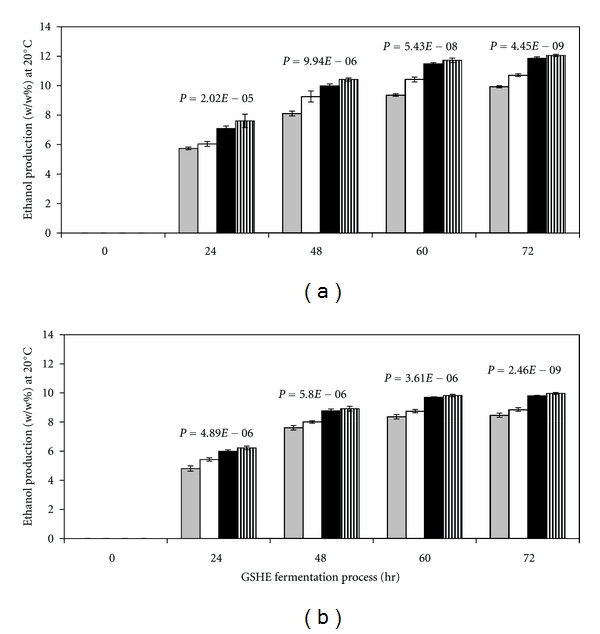
Ethanol yield (based on CO_2_ release) profile of GSHE, under yeast fermentation process at 32 ± 2°C at various GSHE dosage (kg/MT of grain): 1.5 (grey); 2.0 (white); 2.5 (black); 3.0 (striped) when (a) Indian broken rice; (b) Indian pearl millet was used as a raw material. The values represent means ± S.D. of three experimental studies. The *P* value represents between all GSHE dosages at 24 hrs intervals.*P*-value was found 0.04, 0.03, 0.18, and 0.22 in case of Indian broken rice at 24, 48, 60, and 72 hr, respectively, while performing ANOVA of 0.25 and 0.3 kg GSHE dosage, whereas in case of pearl millet, *P*-value was found 0.03, 0.02, 0.29, and 0.35 at 24, 48, 60, and 72 hr, respectively.

The ethanol production (% w/w at 20°C) was calculated based on weight loss or CO_2_ released. Increasing concentration of GSHE with an increase in ethanol production (% w/w at 20°C) was observed with Indian broken rice (Figure 1(a)) and pearl millet (Figure 1(b)) fermentation feedstocks. Furthermore, ethanol yield was found to be maximum at a concentration of 2.5 kg per MT of grain when Indian broken rice (Figure 1(a)) and pearl millet (Figure 1(b)) was used as fermentation feedstocks, but further increasing GSHE concentration at 3.0 kg per MT of grain did not have much impact in enhancing the ethanol production as ethanol yield differences were observed between GSHE dosage of 1.5 and 2.5 kg per MT of grains (Figures 1(a) and 1(b)). The ANOVA has been performed for ethanol production in respect to different dosages (1.5 to 3.0 kg) of GSHE per MT of grain at 24 hr intervals (Figure 1(a)), it has been observed that the *P*-value was found to be less than 0.05, which indicates that there is a difference in ethanol production between the GSHE dosages used in both the feedstocks. However, while making ANOVA between 2.5 and 3.0 kg GSHE dosage per MT of grain, it has been observed that in initial the 48 hr of yeast fermentation process there were significant differences (*P* < 0.05) in ethanol production, but in later stage of the fermentation cycle, there was no any significant difference (*P* > 0.05) in ethanol production observed. Similar statistically studies have been reported by Gohel et al. [31] in strain improvement of *Pantoea dispersa* in the chitinase production. Moreover, considering the economical stand point of industrial scale and results obtained in the lab-scale studies for ethanol production versus GSHE dosages, GSHE 2.5 kg per MT of grain concentration should be considered a maximum dosage for an industrial scale ethanol production.

### 3.3. pH Profile of Fermentation Medium Processed at Various GSHE Concentrations under Yeast Fermentation Conditions

**Figure 2:**
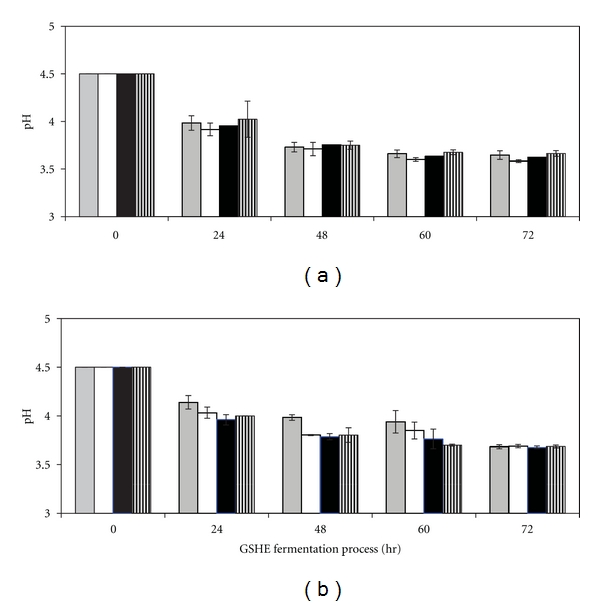
pH profile of GSHE, under yeast fermentation process at 32 ± 2°C at various GSHE dosage (kg/MT of grain): 1.5 (grey); 2.0 (white); 2.5 (black); 3.0 (striped) when (a) Indian broken rice; (b) Indian pearl millet was used as a raw material. The values represent means ± S.D. of three experimental studies.

Yeast fermentation for the ethanol production at pH of 4.0–4.5 is the routine practice to control contaminating bacteria in an industrial scale process [32]. pH of the fermentation medium was also monitored in each concentration of GSHE under yeast fermentation conditions using Indian broken rice (Figure 2(a)) and pearl millet (Figure 2(b)) as fermentation feedstocks. The pH of fermentation medium was found to be decreased from 4.50 to average 3.69 in each experimental study of Indian broken rice and pearl millet feedstocks. It has been reported that decreasing pH during yeast fermentation is due to CO_2_ formation [3]. Decreasing pH may also be due to accumulation of organic-free nitrogen formed by FERMGEN (acid fungal protease) during the GSHE process. The released nitrogen is taken up by the yeast to produce H^+^ ions which results in a gradual decrease in pH of the fermentation medium. This kind of phenomenon has also been demonstrated by Castrillo et al. [33] as the assimilation of one ammonium mole by yeasts leads to the release of one H^+^ mole into the solution. In further support of our study, it has also been shown that between 40 and 160 hr of grape fermentation, ethanol concentration increases in the medium, which may explain the decrease in pH during this period [34].

### 3.4. Ethanol Yield after Distillation

A final ethanol yield was also calculated at the end of yeast fermentation (in 72 hr cycle) through the distillation process. Distilled ethanol yield was estimated by using an alcometer and the readings (%, v/v) were further calibrated at 20°C. Fermentation containing 25% dry solid of Indian broken rice having 68.45% starch resulted in ethanol yields of 11.23 ± 0.08, 11.53 ± 0.10, 11.93 ± 0.06, and 12.09 ± 0.07% v/v at 20°C in 72 hr of yeast fermentation when GSHE was used at concentrations of 1.5, 2.0, 2.5, and 3.0 Kg/MT of grain, respectively, along with 0.2 Kg of FERMGEN per MT of grain. With Indian pearl millet of 25% dry solid having 60% starch with the same enzymes concentrations and experimental conditions resulted in 9.60 ± 0.09, 10.03 ± 0.05, 10.46 ± 0.06 and 10.48 ± 0.04% v/v at 20°C ethanol yield was observed, respectively. Furthermore, based on these distilled ethanol (%, v/v at 20°C) values, ethanol recovery was also calculated in terms of liters per MT of the grain (Table 1) considering the fact that this technology is not only limited to use for fuel ethanol production but also can be used for potable purposes. The ANOVA has been performed for ethanol yield (liter per MT of grain) at various GSHE dosages (1.5 to 3.0 kg per MT of grain). It has been observed that there is a significant difference (*P* > 0.05) in ethanol yield between 1.5 to 2.5 kg GSHE dosages per MT of grain for both the feedstocks (Table 1). However, further ANOVA was compared between GSHE dosages 2.5 to 3.0 kg per MT of grain for both the feedstocks that indicates that the ethanol yield differences are not significant (*P* > 0.05) between 2.5 and 3.0 kg GSHE dosage per MT of grain for both the feedstocks. Henceforth, 2.5 kg per MT of GSHE dosage should be considered a maximum dosage for both the feedstocks in ethanol production through no-cook process under yeast fermentation conditions.

In comparing the two feedstocks grains, ethanol production was observed to be higher with broken rice than with pearl millet, probably due to higher starch content (broken rice had a starch content of 68.45% while pearl millet had 60% starch). Our research study was designed to examine both substrate grains in order to verify that the utility of the no-cook process technology is not limited to Indian broken rice feedstock but applicable to Indian pearl millet feedstock, which is economically more viable for the ethanol industry in India. Sharma et al. [35] reported a 9.10% v/v ethanol yield in GSHE-treated Amioca starch (100%) having 15% dry solids, under the yeast fermentations conditions. Gibreel et al. [3] reported the very high gravity (VHG) fermentation of a hulled variety of barley (with starch content of 59.9%), which yielded an ethanol concentration of 14.87 ± 0.06% on using a pretreatment step prior to GSHE process. It has been reported that the GSHE process with Chinese rice under yeast fermentation conditions yielded 430 to 470 L ethanol per MT, compared to the conventional process with the same substrate which yielded 380 to 400 L of ethanol (http://www.google.co.in/url?sa=t&rct=j&q=http%3A%2F%2Faidaindia.org%2F%20ethanol%20gang%20duan&source=web&cd=1&ved=0CB4QFjAA&url=http%3A%2F%2Faidaindia.org%2Fits08%2Fimages%2Fdate%252020-3-08%2FDone%2520Presentations%2FDr.%2520Duan%2520-%252011.10-11.30.ppt&ei=dz4QT6GPOoanrAf_9dnmAQ&usg=AFQjCNEYJ1e_7lrbU1pk8dUiZRN-e3tFXQ). Duan et al. [36] have reported that the use of phytase along with GSHE for sorghum under yeast fermentation conditions resulted in 380–400 liters ethanol per MT of sorghum. Further, it is documented that addition of phytase along with GSHE under yeast fermentation conditions has further improved the quality of DDGS for animal feed application [36]. However, there is no report on Indian broken rice and pearl millet for the GSHE cold process with or without any pretreatment in ethanol production.

### 3.5. Fermentation Efficiency, Residual Sugar, and Starch Content

In each feedstock, increasing concentration of GSHE resulted in increased fermentation efficiency observed (Table 2). Residual sugar was not detected in any experimental studies. Residual starch was observed in very minimal amount (Table 2).

In comparison to the conventional process, involving higher liquefaction's temperatures, theoretically, 100 gm of starch should produce 56.7 gm of ethanol at the maximum yield, assuming that all starch is completely converted into glucose. In GSHE process in both the feedstocks of present study, 97 to 98% fermentation efficiency was observed (Table 2). However, in practice, only 81 to 90% fermentation efficiency was observed in conventional process [36]. Wu et al. [37] used a three-step conventional process in ethanol production from US pearl millet having 65.30% starch and 25% dry solid concentration. Their process involved liquefaction at 95°C for 45 min followed by 80°C for 30 min, saccharification at 60°C for 30 min, and finally yeast fermentation that resulted in ~11% v/v at 20°C ethanol yield with fermentation efficiency of 90% and residual starch 3.45%. Zhan et al. [38] used the conventional process for US sorghum having 68.8% starch and 25% dry solid concentration and obtained 10.72% v/v ethanol yield with 85.93% fermentation efficiency. It has been reported that this fall in fermentation efficiency in the conventional process is due to the loss of some fermentable sugars as a result of a heat-catalyzed Maillard reaction between amino acids and reducing sugars during jet-cooking process [26]. Furthermore, it has also been reported that presence of soluble sugars like glucose and fructose in broken rice and pearl millet would be ready for utilization by yeast in the no-cook process, while in the conventional process due to higher temperature liquefaction, these free soluble sugars were found to be inactivated because of the Maillard reaction [26]. This inactivated sugars further cannot be utilize by the yeast during the fermentation process [26]. Apart from this disadvantage, the typical conventional process has a process duration disadvantage requiring either three steps (liquefaction, saccharification and fermentation) or two steps (liquefaction and SSF, simultaneous saccharification, and fermentation).

In conventional process, it has been reported that Indian ethanol producer having plant capacity of 110–130 MT of Indian broken rice (68% starch, 28% dry solids) feedstock per day consumed 49.5 MT steam in its liquefaction process to cook Indian broken rice and followed by simultaneous saccharification (SSF), and fermentation process under the yeast fermentation conditions resulted in 10% v/v at 20°C, 410 L ethanol per MT of Indian broken rice with 86% fermentation efficiency (http://www.pcbassam.org/EIAREPORT/EIA_Radiant/2%20Chapter%20(The%20Project).pdf) whilst in case of GSHE process, this 49.5 MT steam per day used in liquefaction could be further saved. This steam savings in GSHE process directly impact in the reduction of overall process cost in ethanol production.

It has also been reported that the biomass of yeast (1.95 kg per 100 kg starch) produced in the no-cook process is less than the conventional process (3.88 kg per 100 kg starch), which indirectly validates the observed increase in conversion efficiency, validating that more sugars were used for ethanol instead of yeast growth [2].

The present investigation reveals the potential of the no-cook process with GSHE (STRAGEN 002) along with acid protease enzyme (FERMGEN) for Indian broken rice and pearl millet feedstocks in ethanol production under yeast fermentation conditions. Furthermore, if this no-cook process replaces the conventional process in ethanol production; there are added benefits of steam savings, lower capital investment/process, and process simplification by reducing unit operations (single step process), these advantages save operational costs, are more environmental friendly, and increase fermentation efficiency.

## Figures and Tables

**Table 1a:** (a)

GSHE dosage (kg per MT of grain)	Yeast fermentation 25% (dry solid) in 72 hr fermentation cycle	
	Liter ethanol per MT of	
	Indian broken rice^a^	Pearl millet^a^
1.5	449.33 ± 3.23	384.13 ± 3.40
2.0	461.33 ± 4.11	401.07 ± 2.01
2.5	477.07 ± 2.41	418.53 ± 2.20
3.0	483.60 ± 2.80	419.33 ± 1.62

^a^Value are means ± SD of three experimental studies.

*ANOVA for ethanol yield at various dosage of GSHE.

**Table 1b:** (b)

Source of variation	Liter ethanol per MT of					
	Indian broken rice			Pearl millet		
	Between group	Within group	Total	Between group	Within group	Total
Sum of square	2155.03	81.92	2236.95	2511.40	46.20	2557.60
Degree of freedom	3.00	8.00	11.00	3.00	8.00	11.00
Mean sum square	718.34	10.24		837.1	5.80	
*F-*statistics	70.15			145.00		
*P-*value	4.36E−06			2.598E−07		

**Table 2a:** (a)

GSHE dosage (kg per MT of grain)	Yeast fermentation 25% (dry solid) in 72 hr^a^			
	Fermentation efficiency* (%)		Residual starch (%)	
	Indian broken rice	Pearl millet	Indian broken rice	Pearl millet
1.5	91.61 ± 0.66	89.28 ± 0.79	0.23 ± 0.02	0.44 ± 0.04
2.0	94.06 ± 0.84	93.22 ± 0.47	0.19 ± 0.01	0.36 ± 0.02
2.5	97.27 ± 0.49	97.28 ± 0.51	0.15 ± 0.01	0.22 ± 0.03
3.0	98.60 ± 0.57	97.47 ± 0.38	0.10 ± 0.01	0.22 ± 0.03

^a^Value are means ± SD of three experimental studies.

*ANOVA for fermentation efficiency at various dosage of GSHE.

**Table 2b:** (b)

Source of variation	Fermentation efficiency in 72 hr					
	Indian broken rice			Pearl millet		
	Between group	Within group	Total	Between group	Within group	Total
Sum of square	89.58	3.41	92.99	135.68	2.50	138.18
Degree of freedom	3.00	8.00	11.00	3.00	8.00	11.00
Mean sum square	29.86	0.43		45.23	0.31	
*F-*statistics	70.15			145.00		
*P-*value	4.36E−06			2.60E−07		
